# Which Factors Are Important for Successful Sentinel Node Navigation Surgery in Gastric Cancer Patients? Analysis from the SENORITA Prospective Multicenter Feasibility Quality Control Trial

**DOI:** 10.1155/2017/1732571

**Published:** 2017-06-15

**Authors:** Ji Yeong An, Jae Seok Min, Young Joon Lee, Sang Ho Jeong, Hoon Hur, Sang Uk Han, Woo Jin Hyung, Gyu Seok Cho, Gui Ae Jeong, Oh Jeong, Young Kyu Park, Mi Ran Jung, Ji Yeon Park, Young Woo Kim, Hong Man Yoon, Bang Wool Eom, Keun Won Ryu

**Affiliations:** ^1^Department of Surgery, Samsung Medical Center, Sungkyunkwan University School of Medicine, Seoul, Republic of Korea; ^2^Department of Surgery, Dongnam Institute of Radiological and Medical Sciences, Cancer Center, Busan, Republic of Korea; ^3^Department of Surgery, Gyeongsang National University, Jinju, Republic of Korea; ^4^Department of Surgery, Gyeongsang National University, Changwon, Republic of Korea; ^5^Department of Surgery, Ajou University School of Medicine, Suwon, Republic of Korea; ^6^Department of Surgery, Yonsei University College of Medicine, Seoul, Republic of Korea; ^7^Department of Surgery, Soonchunhyang University College of Medicine, Bucheon, Republic of Korea; ^8^Department of Surgery, Chonnam National University Hwasun Hospital, Hwasun, Republic of Korea; ^9^Gastric Cancer Center, Kyungpook National University Medical Center, Daegu, Republic of Korea; ^10^Gastric Cancer Branch, National Cancer Center, Goyang, Republic of Korea

## Abstract

**Background:**

We investigated the results of quality control study prior to phase III trial of sentinel lymph node navigation surgery (SNNS).

**Methods:**

Data were reviewed from 108 patients enrolled in the feasibility study of laparoscopic sentinel basin dissection (SBD) in gastric cancer. Seven steps contain tracer injection at submucosa (step 1) and at four sites (step 2) by intraoperative esophagogastroduodenoscopy (EGD), leakage of tracer (step 3), injection within 3 minutes (step 4), identification of at least one sentinel basin (SB) (step 5), evaluation of sentinel basin nodes (SBNs) by frozen biopsy (step 6), and identification of at least five SBNs at back table and frozen sections (step 7).

**Results:**

Failure in step 7 (*n* = 23) was the most common followed by step 3 (*n* = 15) and step 6 (*n* = 13). We did not find any differences of clinicopathological factors between success and failure group in steps 1~6. In step 7, body mass index (BMI) was only the significant factor. The success rate was 97.1% in patients with BMI  <  23 kg/m^2^ and 80.3% in those with BMI ≥ 23 kg/m^2^ (*P* = 0.028).

**Conclusions:**

Lower BMI group showed higher success rate in step 7. Surgeons doing SNNS should be cautious when evaluating sufficient number of SBN in obese patients.

## 1. Introduction

Although radical gastrectomy with D2 lymph node (LN) dissection has been the standard surgical treatment for advanced gastric cancer, it seems to be an overly invasive treatment for patients with early gastric cancer (EGC) because EGC shows limited LN metastasis and excellent survival. Considering that D2 gastrectomy decreases the quality of life (QOL) of patients, sentinel lymph node navigation surgery (SNNS) is an appealing option and has become the new paradigm in EGC treatment [1, 2].

Sentinel LN (SN) is the first site of metastasis through the lymphatic drainage pathway from the primary tumor, and it is well established in breast cancer and melanoma. Also, the concepts of SN lymphatic basin and drainage are applicable in gastric cancer [3]. Several surgeons in Japan and Korea have sought to develop surgical strategy for gastric cancer based on SN status and the concept of sentinel basin dissection (SBD) [1, 4]. Limited gastric resection, such as wedge or segmental resection, and avoidance of unnecessary LN dissection in SNNS must be applicable enough for EGC without SN metastasis [4, 5]. However, the skip, transversal, and micrometastasis of SNs are critical points for using SNNS in clinical practice [6, 7].

In Korea, some surgeons, endoscopists, pathologists, and nuclear radiologists who were interested in SNNS for EGC treatment planned a multicenter phase III trial comparing conventional laparoscopic gastrectomy to laparoscopic SN biopsy with limited gastrectomy in clinical stage Ia gastric cancer patients [8]. Before starting the phase III study, a quality control study was done to qualify the institutions participating in the phase III trial and to standardize surgical procedures [9]. In the quality control study, the protocol for successful SN biopsy consisted of 7 critical steps, which should be performed by endoscopists, surgeons, and pathologists. Success was defined as the accurate execution of all 7 steps, and a minimum of 10 cases of success at each institution achieving this benchmark could participate in the subsequent phase III trial. In this quality control study, laparoscopic SBD was shown to be feasible and sufficiently sensitive for detecting metastatic LNs [9].

For the successful SNNS, high detection rate and low false-negative rate are indispensable in EGC patients. However, few studies have prospectively assessed the factors that are important for successful SNNS. The present study addressed this issue based on the results of the aforementioned quality control study. The failure rate and causes of failure at each step were determined and the clinicopathological features of patients with successful and unsuccessful outcomes were compared. The data should improve the success of SNNS.

## 2. Methods

The protocol of this study was previously described in detail [9] and so is summarized in this report.

### 2.1. Patients

This study enrolled 20- to 80-year-old gastric cancer patients with clinical stage T1-2N0M0 according to the American Joint Committee on Cancer (AJCC) 7th edition [10]. The longest tumor diameter was less than 4 cm under endoscopic measurement, and the tumor was located at least 2 cm away from the pylorus or cardia. Patients with absolute endoscopic submucosal dissection (ESD) indications were excluded. Seven institutions participated in this quality control study. Data of all patients were preoperatively registered in a central data center (eVelos System; Velos, Inc., Fremont, CA). All patients provided written informed consent before the surgery and this study was approved by the local institutional review boards of all participating institutions.

### 2.2. Laparoscopic SBD Procedures and Histologic Examination

**Figure 1:**
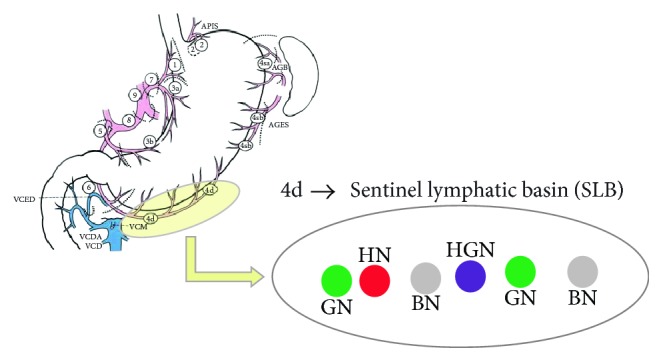
Definition of sentinel basin nodes. GN: green node, HN: hot node, HGN: hot and green node, and BN: basin node.

**Figure 2:**
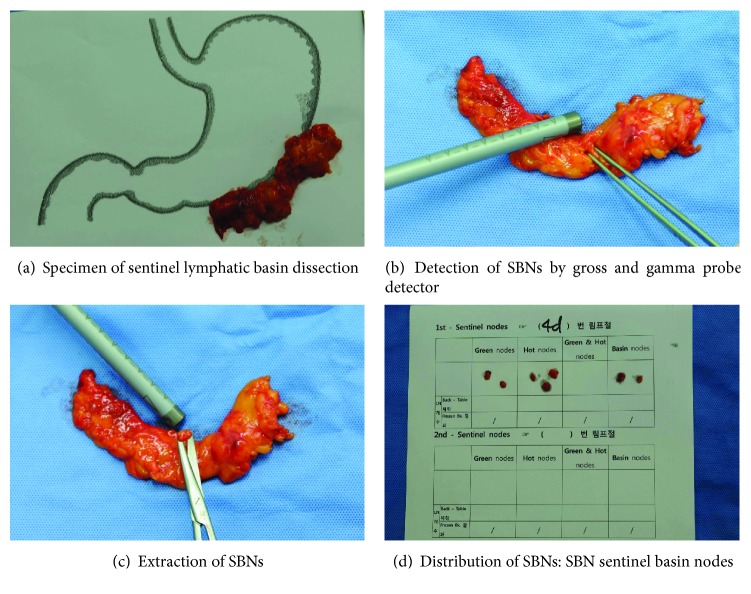
Harvest procedure of sentinel basin nodes at back table.

A mixture of indocyanine green (IGC; Diagnogreen1, Daiichi-Sankyo Co. Ltd., Tokyo, Japan; 2 mL, 5 mg) and radiolabeled human serum albumin (Tc99m-HSA; 2 mL, 0.1 mCi/mL) was used as a tracer to detect SNs. A 4 mL volume of the dual tracer was injected into the submucosal layer in four quadrants of the primary tumor (1 mL at each quadrant) via an intraoperative endoscopic approach. Fifteen minutes after injection of the endoscopic tracer, stained lymphatics and LNs were identified. At the same time, a handheld laparoscopic gamma probe was used to detect the radioactive SNs. Sentinel basins (SBs) containing SNs were carefully dissected and retrieved from the surgical field. The harvested SBs were dissected to isolate LNs at a back table. All the isolated LNs from the SBs were defined as sentinel basin nodes (SBNs). In other words, SBNs mean the whole LNs within the SB, which were classified into 4 kinds, hot node (HN), green node (GN), both hot and green nodes (HGN), and basin node (BN), as described in our previous report [9].  Figure 1() explicates the definition of SBNs. The GN means the only uptake of ICG without Tc99m-HSA, HN does only 99mTc-HAS uptake without ICG, and HGN does concurrent uptake of ICG and 99mTc-HSA. The BN means nonsentinel nodes within the same SB. In other words, LNs were retrieved from the SBs but neither green nor hot were defined as BNs. All SBNs were extracted at back table during surgery and sent to the pathologist for intraoperative frozen sectioning.  Figure 2() shows the procedure of SBNs harvest at back table. LNs harvested from the SBs were histologically examined intraoperatively by hematoxylin and eosin (H&E) staining using one representative cut plane of a frozen section. The standard gastrectomy with lymphadenectomy of D1+ or more was carried out after performing SBD in all patients according to the Japanese gastric cancer treatment guidelines [11]. After the conventional surgery was completed, the remaining LNs were classified into each LN station according to the anatomical definition of the Japanese guidelines and sent for permanent pathologic examination by H&E staining [12].

### 2.3. Checklist

The completion of 7 critical steps to assess the adequacy of the procedure to be performed by endoscopists, surgeons, and pathologists was regarded as a success. Some of steps were checked overlapped as failure depending on the cases. Step 1 verifies that the tracer is injected at the submucosal layer by intraoperative esophagogastroduodenoscopy (EGD). Step 2 verifies tracer injection at four sites by intraoperative EGD. Step 3 verifies the absence of intraluminal or extraluminal leakage of tracer during the injection by intraoperative EGD. Step 4 verifies injection of the tracer within 3 minutes from the first to fourth injection by intraoperative EGD. Step 5 verifies that at least one SB is identified during the laparoscopic surgery. Step 6 is the evaluation of the SBN by intraoperative frozen biopsy. Step 7 is the identification of the SBNs at least five in the back table and frozen sections. To succeed step 7, we should get five or more SBNs in both back table extraction procedure and intraoperative frozen section test. A minimum of 10 cases of success for all 7 steps were needed at each institution for participation in the subsequent phase III to be qualified in this study.

### 2.4. Evaluations

We evaluated the clinicopathological features of 108 patients including accumulation of case numbers (learning curve), age, gender, body mass index (BMI), tumor locations, number and location of SBN, tumor size, histology, and tumor stage in association with success or failure at each step of checklist. SPSS software version 18.0 for Windows (SPSS, Chicago, IL) was used for all statistical analyses. For nominal values, chi-squared or Fisher's exact tests were used, also logistic regression was used for multivariate analysis.

## 3. Results

### 3.1. Failure Causes in All Institutions

Table 1 shows the number of failure cases in each checklist at the 7 participating institutions. Among 108 cases, we experienced 23 failure cases in this quality control trial. Thus, the success rate was 78.7% (85/108). To achieve 10 case completions of all checklists, 13~20 cases were required. Failure in step 7 (*n* = 23) was most common followed by steps 3 (*n* = 15) and 6 (*n* = 13).

### 3.2. Clinicopathological Factors in Association with Identification of at Least 5 SBNs (Step 7)

No differences were evident in clinicopathological factors between success and failure group in steps 1~6 (data not shown). The learning curve did not affect the success rate in all 7 steps. Because insufficient number of SBN was the most important reason in failure cases, we focused on step 7 (identification of at least five SBNs at back table and at frozen biopsy). Among all 108 cases, ninety-five patients finished successfully on step 6, which is the evaluation by intraoperative frozen biopsy, and consequently they moved into step 7. Therefore, we divided ninety-five patients into two groups: step 7 success and failure cases. Learning curve, age, gender, tumor location, number of SBs, SBs location, LN station of SBs, tumor size, histology, and pT and pN status were not related to the status of success or failure in the identification of at least five SBNs (Table 2). The BMI of patients was the only significant factor for success in step 7. The success rate was 97.1% and 80.3% in patients with BMI < 23 kg/m^2^ and ≥23 kg/m^2^, respectively. Also, the failure rate was 2.9% and 19.7% in patients with BMI < 23 kg/m^2^ and ≥23 kg/m^2^ at step 7, respectively (*P* = 0.028). In patients with higher BMI (BMI ≥ 23 kg/m^2^), the odds ratio of failures in step 7 was 8.082 and 95% confidence interval was 1.002~65.156. Also in multivariate analysis, using factors of *P* < 0.25, the BMI of patients significantly affect the rate of failure (*P* = 0.042).

### 3.3. Effect of Patient BMI and SBD Experience on Successful SBN Evaluation according to the Checklist Step 7

**Figure 3:**
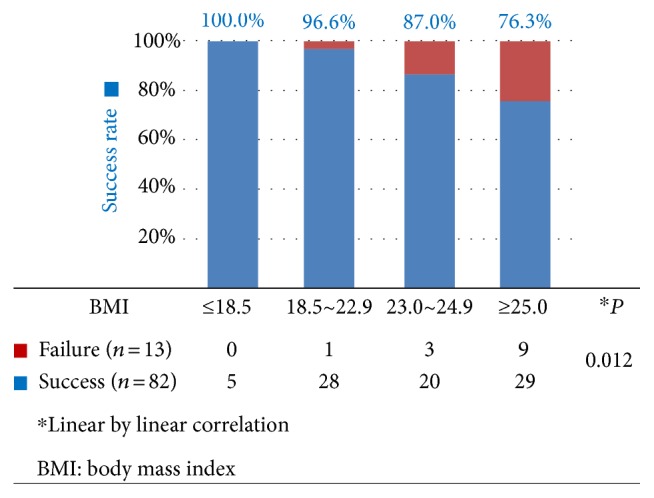
Effect of patient BMI on the identification of at least 5 sentinel basin nodes (step 7 in checklist).

When BMI of patients were divided into four groups according to the criteria of Korean society for study of obesity, the identification of at least 5 SBNs was successful in 100.0% of BMI < 18.5 patients (underweighted), 96.6% in BMI 18.5~22.9 patients (normal range), 87.0% in BMI 23.0~24.9 patients (overweight), and 76.3% in BMI ≥ 25.0 patients (obesity), respectively (Figure 3). The lower BMI group showed higher success rate in the identifications of at least 5 SBN evaluations at back table and frozen section; the rate exhibited a significant linear by linear correlation (*P* = 0.012). In the first half of the cases considering learning curve, the success rate did not statistically differ with differing BMI (*P* = 0.182) (Table 3). However, in the second half of the cases, the failure rate was higher in patients with elevated BMI, with successful SBN evaluation having showed a significant linear by linear correlation with BMI (*P* = 0.029).

## 4. Discussion

A previous multicenter prospective clinical trial of SN biopsy in Japan demonstrated that SN mapping for gastric cancer with dual tracer method is a safe and feasible procedure [1]. In Korea, the SENORITA (Sentinel Node Oriented Tailored Approach) study group performed a quality control trial to achieve optimal outcomes in the subsequent multicenter phase III trial [9]. Based on the results of this feasibility study, no patients were revealed to have metastatic node on permanent H&E staining examination after negative findings on frozen biopsy tests and vice versa. The method of intraoperative pathologic examination used in present study had sensitivity and accuracy rate of 100.0% in terms of detecting metastatic LN when compared to permanent pathologic results by H&E examination. In reference, laparoscopy-assisted distal gastrectomy was the most commonly performed surgical procedure after SBD (101/108, 93.5%) in quality control trial. Eight patients (7.4%) experienced postoperative complications in early postoperative period (<30 days), but none were directly related to additional procedure of SBD.

Seven critical steps to assess the adequacy SBN evaluation procedure were decided based on previous studies [1, 2, 4, 13]. Because the learning curve for identification of SBN required 26 cases to reach a 95% success rate in cumulative sum analysis, the benchmark in the quality control study was a minimum of 10 cases in each institution; this number of successes was judged adequate to overcome the initial learning curve for sentinel node navigation surgery for gastric cancer and for inclusion of an institution in the multicenter phase III trial [9, 14]. As mentioned in the quality control study, the authors expected that about 30 cases would be needed to achieve 10 successful cases. However, counter to this expectation, an average of 15.4 cases (a total of 108 cases in 7 institutions) was required [9]. More than half of all institutions had already individual experience more than 10 cases with SBN evaluation. Sharing of these experiences was important in decreasing the learning curves.

Finding at least 5 LNs at back table and frozen biopsy is a critical step for determining success or failure in SNNS. A meta-analysis found that sensitivity of SN biopsy in gastric cancer was significantly related to the number of harvested SNs [4]. The pooled estimate for ≤4 and >4 SNs had a sensitivity of 82.3% and 92.6%, respectively; the difference was significant. However, the sensitivity of SN number >5 showed no significant difference compared with ≤5 and this was the same with the cutoff value of 6 SN number. Because the absolute sensitivity of SN number >5 or >6 was not improved compared to >4, the identification of at least 5 SNs was a critical step in this quality control study to decrease false negative rate.

The lower BMI group showed significantly higher success rate in detection at least 5 SBNs at back table and frozen section, with a linear by linear correlation evident. Surgeons can be challenged to retrieve sufficient SBNs when confronted by a fatty dense abdominal cavity. Similarly, pathologists can find it difficult to identify fatty SBNs. Although other authors reported that increased fat contents have little effect on the number of LNs retrieved in laparoscopy-assisted distal gastrectomy [15], excess fat tissues may limit accessibility to LNs located deep in the adipose tissue around abdominal vessels [16]. In these studies, overweight was associated with a lower likelihood of LN metastasis; the excessive fat tissues around the lymphatics might disturb the lymphatic flow as well as tumor cell migration. Also, visceral obesity might disturb the tracer flow through the lymphatics and decrease the sensitivity of SBN detection.

The success rate was 100% (5/5) for underweighted patients whose BMI was ≤18.5 kg/m^2^ and was markedly decreased to 76.3% (29/38) in obese patients with a BMI ≥ 25.0 kg/m^2^ (Figure 3()). On the other hand, even in second half of the cases, the success rate among patients with BMI ≥ 25.0 kg/m^2^ was only 70.6% (12/17), while it was 100% (3/3) in patients with BMI ≤ 18.5 kg/m^2^ (Table 3). Considering these results, lower BMI is important for achieving successful results of SBD. Although more experience in SBD can reduce failure rate, institutions starting SNNS should be cautious in obese patients following early implementation of SNNS.

The SENORITA multicenter, phase III, randomized control trial for individualized function-preserving gastrectomy including gastric wedge resection, segmental gastrectomy, and intraoperatively endoscopic submucosal dissection with SBD for T1N0 gastric cancer is currently underway in Korea [8, 17]. In the phase III trial, the execution of all 7 checklist steps and also lower BMI of patients are not mandatory, and the operator decides the feasibility of function-preserving gastrectomy during the operation by considering suitability and possibility of successful SBN evaluation, because the participating institutions were already qualified through the quality control study. We will report the results of phase III trial as soon as possible after the trial completion.

In conclusion, patients with BMI < 23 kg/m^2^ had a significantly higher success rate in the detection of at least 5 SBNs at back table and frozen section, even as the personnel became more experienced in the detection procedure, and BMI affected the success of SBD. Surgeons starting SNNS should be cautious in obese patients concerning the retrieval of sufficient number of sentinel LNs and successful SBN evaluation.

## Figures and Tables

**Table 1:** Number of failure cases at each checklist.

Institution	Checklists						
	Step 1	Step 2	Step 3	Step 4	Step 5	Step 6	Step 7
A			4	1		2	2
B			3		1	2	5
C			1	2			1
D	1	1	2	1	2	2	4
E			3		4	3	3
F							3
G	2	2	2	2	4	4	5
Total number of failure cases at each checklist	3	3	15	6	11	13	23

Step 1. Tracer injected at submucosal layer by intraoperative EGD—yes or no. Step 2. Tracer injected at 4 sites by intraoperative EGD—yes or no. Step 3. Intraluminal or extralumial leakage of tracer occurred during the injection by intraoperative EGD—yes or no. Step 4. Tracer injected within 3 minutes from 1st injection to 4th injection by intraoperative EGD—yes or no. Step 5. At least 1 SLB was identified during the laparoscopic surgery—yes or no. Step 6. SBNs (HN, GN, HGN, and BN) were evaluated by intraoperative frozen biopsy—yes or no. Step 7. SBNs (HN, GN, HGN, and BN) were identified at least 5 at the back table and frozen section—yes or no. EGD: esophagogastroduodenoscopy; SLB: sentinel lymphatic basin; SBN: sentinel basin node; HN: hot node; GN: green node; HGN: hot and green node, BN: basin node.

**Table 2:** Clinicopathological factors associated with at least 5 SBN evaluations at back table and frozen section (step 7 in checklist).

	Success in step 7 (*n* = 82)	Failure in step 7 (*n* = 13)	^∗^ *P*	^∗∗^ *P*
Learning curve			0.734	
First half cases	40 (85.1%)	7 (14.9%)		
Last half cases	42 (87.5%)	6 (12.5%)		
Age (years)			1.000	
<60	53 (86.9%)	8 (13.1%)		
≥60	29 (85.3%)	5 (14.7%)		
Gender			0.558	
Male	37 (84.1%)	7 (15.9%)		
Female	45 (88.2%)	6 (11.8%)		
BMI (kg/m^2^)			0.028	0.042
<23.0	33 (97.1%)	1 (2.9%)	Odds ratio = 8.082	Odds ratio = 10.322
≥23.0	49 (80.3%)	12 (19.7%)	95% CI = 1.002~65.156	95% CI = 1.082~98.437
Longitudinal tumor locations			0.319	
Lower 1/3	45 (81.8%)	10 (18.2%)		
Middle 1/3	36 (92.3%)	3 (7.7%)		
Upper 1/3	1 (100.0%)	0 (0%)		
Circumferential tumor locations			0.059	
Lesser curvature	24 (92.3%)	2 (7.7%)		
Greater curvature	27 (90.0%)	3 (10.0%)		0.881
Anterior wall	21 (91.3%)	2 (8.7%)		0.812
Posterior wall	10 (62.5%)	6 (37.5%)		0.056
Number of sentinel basin			0.313	
1	32 (82.1%)	7 (17.9%)		
≥2	50 (89.3%)	6 (10.7%)		
Sentinel basin location			0.666	
Lesser curvature	27 (90.0%)	3 (10.0%)		
Greater curvature	28 (82.4%)	6 (17.6%)		
LC + GC	27 (87.1%)	4 (12.9%)		
LN station of sentinel basin			0.593	
N1 group (LN numbers 1~6)	74 (85.1%)	13 (14.9%)		
N2 group (LN numbers 7, 8a, 9, and 11d)	8 (100.0%)	0 (0%)		
Endoscopic tumor size (cm)			0.156	0.054
≤2	36 (92.3%)	3 (7.7%)		
>2	46 (82.1%)	10 (17.9%)		
Histology			0.210	0.105
Tubular adenocarcinoma	54 (90.0%)	6 (10.0%)		
Signet ring cell carcinoma	26 (78.8%)	7 (21.2%)		
pT			0.644	
T1	73 (86.9%)	11 (13.1%)		
T2~4	9 (81.8%)	2 (18.2%)		
pN			1.000	
N0	72 (85.7%)	12 (14.3%)		
N1~3	10 (90.9%)	1 (9.1%)		

^∗^Chi-square test or Fisher's exact test. ^∗∗^Multivariate analysis, using factors of ^∗^*P* < 0.25. BMI: body mass index; CI: confidence interval; LN: lymph node.

**Table 3:** Effect of patient BMI and sentinel basin dissection experience in checklist step 7.

BMI	First half cases			Last half cases		
	Success (*n* = 38)	Failure (*n* = 7)	^∗^ *P*	Success (*n* = 42)	Failure (*n* = 6)	^∗^ *P*
<18.5	2 (100.0%)	0 (0%)	0.182	3 (100.0%)	0 (0%)	0.029
18.5~22.9	10 (100.0%)	0 (0%)		18 (94.7%)	1 (5.3%)	
23.0~24.9	11 (78.6%)	3 (21.4%)		9 (100.0%)	0 (0%)	
≥25.0	17 (81.0%)	4 (19.0%)		12 (70.6%)	5 (29.4%)	

^∗^Linear by linear correlation. BMI: body mass index.

## Additional Points

*Synopsis*. This quality control study prior to a phase III trial of sentinel lymph node navigation surgery showed that lower BMI was important for the retrieval of sufficient number of sentinel nodes.
